# Futurist Art: Motion and Aesthetics As a Function of Title

**DOI:** 10.3389/fnhum.2016.00201

**Published:** 2016-05-17

**Authors:** Stefano Mastandrea, Maria A. Umiltà

**Affiliations:** ^1^Department of Education, University of Roma TreRoma, Italy; ^2^Department of Pharmacy, University of ParmaParma, Italy; ^3^Department of Art History and Archeology, Columbia UniversityNew York, NY, USA

**Keywords:** art perception, dynamism, futurism, titles, aesthetic evaluation

## Abstract

Very often the titles of Futurist paintings contain words denoting movement in order to satisfy their artistic poetic focused on motion and velocity. The aim of the present study is to investigate the reported dynamism and aesthetic quality of several Futurist artworks as a function of their title. Ten Futurist artworks with a movement-related word in the title were selected for this study. The titles were manipulated, resulting in four conditions for each painting: the “original title” with the movement word; an “increased” title in which an adjective was added in order to intensify the sense of dynamism; a “decreased” title, in which the movement word was eliminated; no title. Participants evaluated the movement suggested by each painting in the four different title conditions, rated their beauty and reported how much they liked the work. Results showed that the manipulation of the title had an effect on the reported movement: compared to the others, paintings presented with the “original” and with the “increased” title received significant higher movement scores. Of interest, beauty did not differ across conditions, but liking was higher for the conditions with more movement. Lastly, positive correlations between the quantity of perceived movement and aesthetic evaluation were found. From the present results it can be concluded that Futurists attributed much relevance to the titles of their artworks in order to effectively increase the expression of the movement represented.

## Introduction

Since the time of ancient art, representing movement in a static 2D medium has been a challenge for artists. The Greek statue of the *kouros* (dated to the Archaic period, around the seventh century B.C.E.) with the left leg bent forward is an early representation of dynamism via the breaking of the bilateral symmetry of the body. In painting, sculpture, and photography, the viewer has the illusion that what he/she sees is a real movement. Images convey the impression of dynamism through several structural characteristics and regularities that human beings are able to grasp. Arnheim (1974) argued the dynamism perceived does not always derive from intrinsic characteristics of motion of the object (running man, waterfall, etc.), but by compositional features such as asymmetry, which can be considered the result of lines and shapes with an indication of direction. For example, a representation of an object with an oblique position and a wedge-shaped configuration suggests motion to most people; while the same object will appear static and rigid if depicted without satisfying these perceptual conditions.

A recent art style that more than others considered the representation of movement as a central issues of its poetics was the Futurist Avant-garde of the first two decades of the twentieth century. Futurists exalted the modern age and industrialization, with its new mechanical tools and machinery, offering optimism about technology and progress. According to this view there was the need for a clear cut with the tradition and with the art of the past. Futurist artists such as Marinetti, Balla, Boccioni, Carrà, Russolo and Severini tried to find new ways to present this new époque. They aimed to represent movement, dynamism and velocity. In the Futurist Manifesto (Marinetti, 1909), some excerpts focused on motion and speed: “We declare that the splendor of the world has been enriched by a new beauty: the beauty of speed. A racing automobile with its bonnet adorned with great tubes like serpents with explosive breath…a roaring motor car which seems to run on machine-gun fire, is more beautiful than the Victory of Samothrace.” With this declaration, the adventure of one of the most famous Avant-garde movements, the Futurism, started officially. Futurists explored many forms of art such as painting, sculpture, music, architecture, dance, photography and cinema. Futurists used different techniques for the representation of motion in a static image. In the Technical Manifesto of Futurist Painting (1910) they wrote “On account of the persistency of an image upon the retina, moving objects constantly multiply themselves; their form changes like rapid vibration, in their mad career. Thus running horse has not four legs, but twenty, and their movements are triangular.” Blur can be considered efficient at communicating motion in a relatively long-exposure photograph (Cutting, 2002). Important examples of blur and multiple images are Giacomo Balla's painting *Dynamism of a Dog on a Leash* (1912), and the photodynamism artwork by Anton Giulio Bragaglia, *Change of Position* (1911). Balla's masterpiece, *Girl running on the balcony* (1912) reveals the influence of the French physiologist Etienne Marey's chronophotography, in which the vectors of the movement are captured, as in his study *Human Movement* (Marey, 1914).

Probably the simplest way of suggesting motion was diagonal lines with acute vertices, and action lines. Depicting interprenetating shapes and objects of different colors give a clear impression of movement in Giacomo Balla's *Iridescent Compenetration* (1913).

To capture dynamism, Futurism introduced an “Aesthetic of velocity” by abolishing single-focus traditional perspective in favor of multiple images, taken in succession, like stroboscopic images, overlapping them so we see all the images at once (Mastandrea, 2012). In sum, Futurists wanted to depict in a single frozen image the motion formed by an unfolding, continuous sequence of movements.

Futurists images are often accompanied by movement-related words in their title, such as dynamism, movement, walking, running, flying, etc. In addition, the title of the artwork *Running automobile, velocity* + *lights* (*Automobile in corsa, velocità* + *luci*, 1912) with the mathematical sign “+,” can be attributed to the “Parole in libertà” (“Words-in-freedom”) by Marinetti. According to Lista (2008) there is another evidence of an original title denoting movement such as *Plasticity* + *noises* + *velocity* (*Plasticità* + *rumori* + *velocità*, 1913-14). We will refer to the 10 Futurist artworks used in this research with the title commonly-accepted having a movement denoting word in it.

In the literature there are some studies showing that the title influences the aesthetic appraisal of the pictures observed. Millis (2001) found that a metaphorical title (Kennedy, 1986), congruent with the content of the painting, leads to greater aesthetic appreciation, compared to the condition with no title or with only a descriptive title. Titles can offer information that enriches comprehension of the artwork. Leder et al. (2006) showed that for short presentations (1 s) descriptive titles increase the understanding of the paintings more than elaborative titles though for longer presentations (10 s), elaborative titles increase comprehension more. In addition, judgment that a painting is “liked” is often faster than the judgment that is “understood” because liking is often based on a rapid affective assessment of features such as symmetry and color, while understanding often requires considerable interpretation of many features (Leder et al., 2006).

Millis (2001) and Leder et al. (2006) investigated the effect of an image's title on liking and the understanding. In the present research we are primarily interested in exploring the expressive quality of dynamism, represented in several Futurist paintings, through the manipulation of the titles to do with movement. As far as we know no studies dealt with this topic.

Given the same images which possess intrinsic and universal features (lines, shapes, direction, etc.) to express motion, the main purpose of our study was to investigate if the presence, modification or absence of the original titles would produce different outcomes in the perception of the motion expressed in the artworks and in their aesthetic evaluation, both in terms of beauty and liking.

The paintings without any title would constitute the baseline of the evaluations. According to our hypothesis, the Futurist paintings with the original title containing a movement-related word should increase the dynamism perceived by beholders when compared to the no title presentation. The modification of the title, strengthening the motion effect by adding a movement-related word, should further increase the dynamism effect; on the contrary, a neutral title without any movement-related word should decrease the perceived dynamism. Finally, the increased perceived dynamism should modulate the aesthetic appraisal of the beholded paintings.

## Methods

### Participants

The sample consisted of 100 young individuals recruited with a public ad; they had no training in art and volunteered to participate to the experiment. The age range was between 18 and 38 (mean age 21.9, SD ± 3.15; *F* = 85, *M* = 15). They had normal or corrected-to-normal vision and were naive about the purpose of the experiment.

### Stimuli

Ten digital images of Futurist paintings containing a word denoting movement in the title were selected. The artworks employed in the experiment were: (1) Giacomo Balla (1912), *Girl running on a Balcony*; (2) Giacomo Balla (1912), *Dynamism of a dog on a leash*; (3) Giacomo Balla (1913), *Flight of swallow*; (4) Giacomo Balla (1913), *Expansion x velocity*; (5) Giacomo Balla (1913), *Automobile* + *velocity* + *light*; (6) Umberto Boccioni (1911), *Running train*; (7) Benedetta Cappa Marinetti (1919), *Velocity of a boat*; (8) Carlo Carrà (1910), *Dynamism of swimmers*; (9) Luigi Russolo (1912-13), *Dynamism of an automobile*; (10) Gino Severini (1912), *Dynamism of a dancer*.

Five images represented biological motion (person or animal) and five images represented mechanical motion (train, car and boat). We selected paintings belonging to the two categories biological (humans or animals) and mechanical (train, car, boat) in order to verify a potential difference in the perception of dynamism. The original title of the paintings was manipulated in three different ways: by adding or subtracting a movement word, and by removing the title. There were, therefore, 4 experimental conditions: (1) “Original” title; for example, *Girl running on the balcony*; (2) “Increased” title, *Girl running fast on the balcony*; (3) “Neutral title”: *Girl on the balcony*; (4) No title: the image was presented alone. The total number of presented stimuli was 40 (see Appendix for a complete list of paintings and titles). The image and the corresponding title were presented simultaneously in the same slide.

The images were high quality colored digital reproductions, scanned from two books (Lista et al., 2008; Lista and Masoero, 2009). The 10 digital images had a rectangular shape, width between 15 and 21 cm. and height between 12 and 15 cm. The area of the stimuli varied, given the original proportions of each painting. The resolution in the display was between 399 and 710 pixels per inch for the height and between 415 and 628 for the width, with 120 dots per inch.

### Measures

Participants were asked to rate each of the 40 stimuli answering to 3 different Likert questions at 11 points, with a scale from 0 to 10, where 0 corresponded to “not at all” and 10 to “very much.” The questions were: (1) “Do you like this image?” (subjective aesthetic appraisal); (2) “Is this image beautiful?” (objective aesthetic appraisal); (3) “Is there some movement in this picture? If yes how much?” (Amount of movement perceived). Given 10 images, 4 titles and 3 questions, participants gave 120 responses. At the end of the experiment two art education questions were asked: first on the artistic training received (using the same 11 points Likert scale) and, second, on the number of visits to museum or art gallery in the last 12 months.

### Procedure

Participants were seated in a comfortable chair, placed in an isolated, dimly lit room, in front of a 19 inch computer monitor used for stimuli presentation and located at a distance of 70 cm from participant's body. Each participant evaluated the 10 images in the 4 experimental conditions (Original, Increased, Neutral, Untitled) for a total of 40 pictures. The presentation order of the stimuli was completely randomized using the software Inquisit 3.

Each participant had a booklet of 41 pages, one page with three questions per image. After the exposure to each stimulus, participants were required to respond to the questions by check-marking the relevant number of the 11 points scale, with no time limit. Participants pressed “enter” to move to the next image and turned to the next booklet page. Once all 40 stimuli were assessed, the 2 art-education questions were asked. The duration of the experiment was about 30 min.

### Statistical analyses

Statistical analyses were performed with Statistica 7.1 software (StatSoft: http://www.statsoft.com/).

The first aim of the study was to assess whether the 4 different titles associated with the same image were able to modify participants' evaluation of the aesthetic appraisal and amount of perceived movement. In order to verify this hypothesis, the score that each participant gave to the 3 questions was entered in a within participants repeated measures ANOVA (*P* < 0.05). The factors entered in the ANOVA were: Type of Question (3 levels: Liking, Beauty, Amount of movement) × Biological/Mechanical Motion (2 levels) × Title (4 levels:, Original, Increased, Neutral, Untitled). *Post-hoc* analysis (Duncan test *p* ≤ 0.05 automatically adjusted for multiple comparisons) was applied on all significant main factors and interactions. The Shapiro-Wilk test was used to check for normal distribution of all variables entered in the ANOVA (W ≥ 0.9, *P* > 0.05).

In addition, we performed the following Pearson correlation analyses: (1) Amount of perceived movement with both Aesthetic Appraisals; (2) Artistic background with both Aesthetic Appraisals, Amount of perceived Movement and number of visits to museums/galleries.

The scores of Aesthetic Appraisals (Liking and Beauty) and Amount of movement were given for the four different Titles (corresponding to the four experimental conditions) while the scores on the art education were requested only once to each participant. Hence, in order to obtain one value for each question for each participant, the scores of Aesthetic Appraisals and Amount of Movement were averaged across conditions.

## Results

The Shapiro-Wilk test showed that all variables were normally distributed (all Ws ≥ 0.98, all *P*s > 0.05).

The results of the repeated measures within participant ANOVA showed a significant main effect of Question [*F*_(2, 198)_ = 22.994, *P* = 0.000] and a significant Question × Title interaction [*F*_(6, 594)_ = 7.5819, *P* = 0.000].

A Duncan *post-hoc* test applied on the significant main effect of Question shows that the score given by participants on the Amount of Movement (mean = 6.102, SE ± 0.139) is the highest when compared with the other two scores on Aesthetic Appraisal (*P*s < 0.000, Liking = 5.26, SE ± 0.151, Beauty = 5.382, SE ± 0.138).

**Figure 1:**
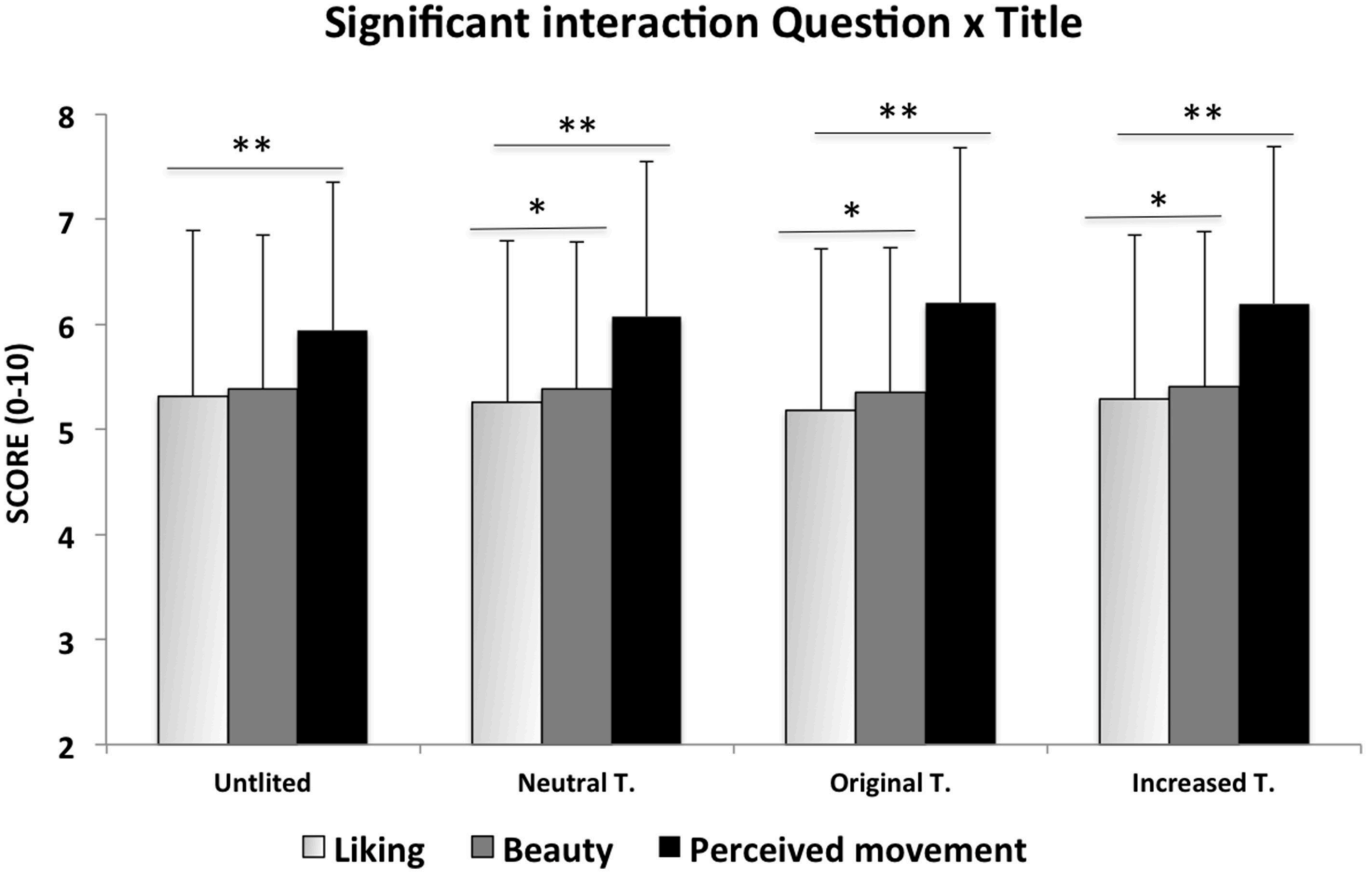
**Results of the ANOVA showing the significant interaction Question × Title**. The asterisks indicate the significant *post-hoc* Duncan test comparisons: ^*^p < 0.005. Only differences among Questions were showed. See the text for differences among different Titles inside each Question. Error bars are SD.

The same *post-hoc* test was also applied on the significant Question × Title interaction (see Figure 1). Beauty was not modulated by the Titles (*P*s > 0.5; UT (Untitled) mean = 5.387, SE ± 0.146; NT (Neutral Title) mean = 5.384, SE ± 0.14; OT (Original Title) mean = 5.357, SE ± 0.136; IT (Increased Title) mean = 5.403, SE ± 0.147), and it received higher scores, when compared with the Liking, for all the Titles except the UT (all *P*s < 0.03).

Differently, the Liking varies in relation with the different Titles (UT mean = 5.315, SE ± 0.158; NT mean = 5.258, SE ± 0.153; OT mean = 5.181, SE ± 0.153; IT mean = 5.289, SE ± 0.155) and, among the Liking scores, the NT and OT were rated significantly lower than UT and IT (*P*s < 0.05).

The Amount of perceived Movement was rated significantly higher than the Liking (*P*s < 0.000) and Beauty (*P*s < 0.000) in all the experimental conditions (UT mean = 5.937, SE ± 0.141; NT mean = 6.071, SE ± 0.147; OT mean = 6.205, SE ± 0.147; IT mean = 6.196, SE ± 0.149). The pattern of modulation of the Amount of Movement in relation with the different titles appears particularly interesting being the UT rated with the lowest (*P*s < 0.005), followed by the NT (*P*s < 0.005), while the IT and OT were rated equally (*P* > 0.5) with the highest scores (*P*s < 0.05).

**Figure 2:**
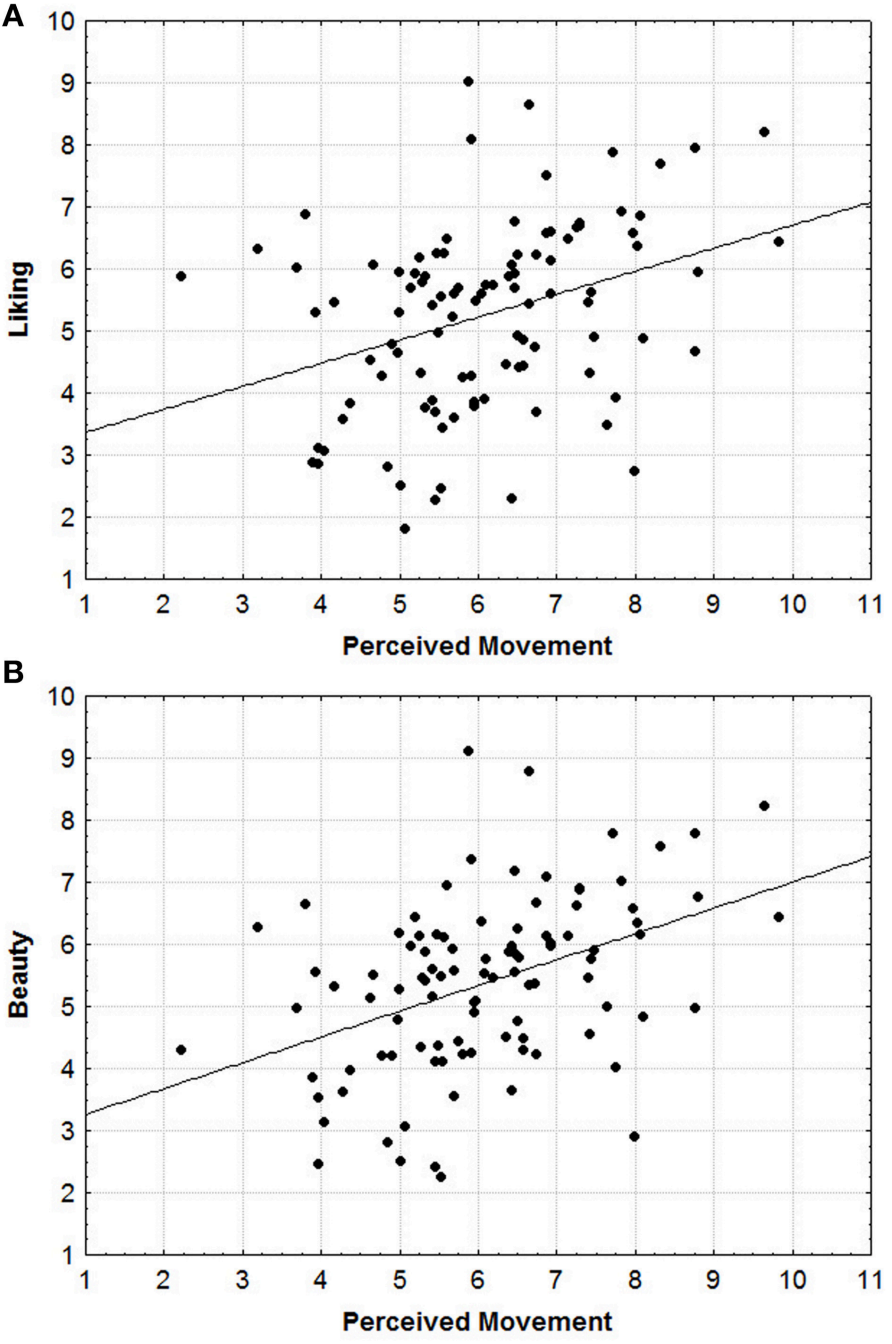
**(A,B)** Scatterplots of the significant correlations between the averaged score of the perceived Amount of Movement with Liking **(A)** and Beauty **(B)**.

The first two correlations performed were aimed to investigate the existence of positive correlations between Amount of Movement and the two different Aesthetic Appraisals. The results confirmed our initial hypothesis showing that the averaged score of the perceived Amount of Movement significantly correlated with both, Liking (*r*^2^ = 0.1175; *r* = 0.342; *p* = 0.000) (see Figure 2A) and Beauty (*r*^2^ = 0.176; *r* = 0.420; *p* = 0.000) (see Figure 2B) Aesthetic Appraisals.

Finally, the Artistic Background of participants significantly correlated with: Amount of Movement (*r*^2^ = 0.115; *r* = 0.339; *p* = 0.000), Liking (*r*^2^ = 0.175; *r* = 0.419; *p* = 0.000), Beauty (*r*^2^ = 0.1802; *r* = 0.424; *p* = 0.000) and number of visits to museums/galleries (*r*^2^ = 0.110; *r* = 0.332; *p* = 0.000).

## Discussion

The discussion will address the three main obtained results: the effect of the manipulation of the title in relation to the perceived amount of movement; differences between Liking and Beauty; correlation between the perceived amount of movement and aesthetic evaluations.

### Effect of the title manipulation

The first interesting finding of our study is that the manipulation of the title has a specific effect on the evaluation of the perceived amount of movement. The paintings presented with the original title (*The girl running on the balcony*) or with the increased title (*The girl running fast on the balcony*) received significant higher scores compared to the paintings presented with no title or with a neutral title (e.g., *The girl on the balcony*, without any terms indicating movement). This finding clearly shows that the idea of movement conveyed by the title (both with the original title term “running,” and with the increased title “running fast”) induces the observer to see a significantly higher amount of the movement depicted. Even though we expected to find a difference, in terms of dynamism perceived, between the original and the increased title (with more dynamism for the increased title), in fact there were no significant differences. Arnheim (1974) stated that the structural and compositional features used by the artist to represent the movement within the subject depicted in a painting have the function to translate the intensity of the physical motion into a pictorial dynamic. Futurists were very successful in representing motion in a static painting. After all one of the main points of the poetics of this Avant-guarde movement (as it was declared in its manifesto of the 1909) was the representation of movement, speed and velocity. It is well known that Futurists payed very much attention in the choice of the title for the artworks representing dynamism: *The girl running on a balcony, Dynamism of a dog on a leash, Running car, Running train, Automobile* + *velocity* + *light* (this last title is a clear Futurist example of using the mathematical sign “+” to link the words), etc. Adding a movement word to the original title was ineffectual. “Running” in the title “Girl running on a balcony” was enough; adding “fast” does not change the reported dynamism. One plausible explanation could be that the extra word here was an adjective. Several studies have shown that the processing of action related verbs elicits automatic activation of the cortical motor system (Pulvermüller and Fadiga, 2010; Pulvermüller, 2013). Action related verbs and movement-related nouns are by themselves able to effectively convey the idea of dynamism; an adjective does not have the power to increase the motion reported.

### Liking and beauty

A relation between these is an important debate in aesthetics. Can Beauty, be an objective feature, different from Liking, seen as subjective? Berlyne (1971) in his new experimental aesthetics (dating back to Fechner, 1876) argued objective characteristics of the object (proportion, symmetry, complexity, etc.) produce appreciation. But liking surely depends on the beholders' characteristics, lay vs., expert, for example. Both cognitive and emotional processing differs idiosyncratically (Millis, 2001; Leder et al., 2006; Mastandrea and Maricchiolo, 2014). Winston and Cupchik (1992), for example, proposed that people with art training evaluate works of art with reference to the artistic style of the composition, while laypeople have a tendency to evaluate works of art according to their own individual feelings and emotions. To this purpose, two questions were asked to participants: “How much they liked each painting” (we refer to this as a subjective aesthetic appraisal) and “How much they found beautiful each painting” (we refer to this as an objective aesthetic appraisal). We understand that the difference between liking and beauty, put it in this term, might be viewed as naïve: the problem is much more extended. Indeed, contemporary art may also arouse aesthetic negative emotions; the aesthetic category of beauty is no longer suitable for contemporary art or it plays only a secondary role. Artworks by Francis Bacon, Lucien Freud, Andres Serrano, and so forth, can be provocative and distressing but also interesting and can be very much appreciated. In our present research, we have taken into consideration (from the broad field of aesthetic) only some basic aspects of the aesthetic appraisal such as Liking and Beauty. These are broad but simple category that lay participants can easily understand. When judging the Beauty of an artwork, one is probably more focused on its intrinsic formal features. In this case beholders likely adopt a sort of psychological distance from their personal and idiosyncratic feelings and aesthetic values (Cupchik, 2002); on the contrary, if beholders have to judge how much they like the artwork, their personal feelings and aesthetic values are paramount. Results showed that Beauty was not modulated by manipulation of the titles, since there were no significant differences among the mean scores, and it received higher scores compared to Liking for all the titles except the presentation with no title. On the contrary, the Liking varied according to the different titles: the conditions with no title and increased title received significantly higher scores than the neutral and the original title conditions. In this last case it is interesting to note that the most liked paintings were those presented with no titles; in other terms, when beholders are completely focused on the impact that structural and compositional features of the painting exert on their subjective feelings.

### Correlation between movement and aesthetic appraisal

As movement ratings increased, both Liking and Beauty increased. As far as we know there are no other studies demonstrating such a relationship between the expressive quality of paintings (dynamism) and their aesthetic evaluation. Arnheim (1949) defined expressions as the psychological correspondence of the dynamic processes taking place in the organization of perceptual stimuli; in other words, the relationship between the stimulating pattern (the dynamics of the visual form) and the expression that it transmits. The possible range of expressive qualities of objects is very wide and dynamism is one of the most important features of an artwork. Paintings can be rated beautiful and appreciated for several aspects related to their intrinsic formal features, the content they convey and for many other reasons; in this present research as motion appraisal increases, the aesthetic appreciation (Liking and Beauty) increases. It seems observers accepted Futurist poetics focused on dynamism and speed.

Further, as art training increased, the movement reported, along with Liking and Beauty ratings, increased. In this vein, liking scores increase with the expertise.

These findings are in agreement with several studies that showed the strong influence of exposure to art and expertise. In particular, among different evaluation parameters the liking score was demonstrated to be significantly influenced by the level of expertise of beholders (Hekkert and van Wieringen, 1996; Kirkand and Freedberg, 2015). The knowledge of art (acquired through art classes at school, visits to museums and galleries, reading art books, etc.) facilitates what Smith and Smith (2006) call *aesthetic fluency*; a process that, in this case, can lead people to better grasp those aspects related to the movement represented in the artwork and to its aesthetic appreciation.

We clearly demonstrated that the title can facilitate not only the appreciation of the artwork (Millis, 2001; Leder et al., 2006), but can also give relevant information about other important characteristics such as the dynamism through which the artist aimed to depict the subject. More in detail, the Futurist artworks selected in this study reached the maximum expression of movement within the original title; stressing the title with a motion word intensifier did not change the results. Speed and velocity were so important to Futurists' poetics that they probably paid much attention to the creation of the title, stressing the motion concept with precise words. We shouldn't forget that Futurism was also a literary movement and the founder Marinetti was a poet and a literate.

A limitation of the study is the laboratory setting with the use of digital reproduction of the original artworks presented to the participants. We believe that additional research involving participants or visitors in the real galleries context should be conducted, bearing in mind that findings could be different. Moreover, the real setting approach with genuine art museum visitors would allow psychology of the arts to get closer to the authentic world of art (Mastandrea et al., 2009).

Let us finally discuss the putative neural bases of the perception of dynamism when beholding static images. A previous neuroimaging study demonstrated the involvement of motion-sensitive extrastriate visual cortex during the observation of static images representing implied motion (Kourtzi and Kanwisher, 2000; Kim and Blake, 2007; Osaka et al., 2010). In sum, these studies highlighted the role of the vision-related cortical areas in the perception of implied motion from static images. It should be added, though, that a series of studies revealed the crucial role, not only of the visual brain, but also of the sensory motor system in abstract art perception. Two electroencephalographic (EEG) experiments showed the activation of the sensorimotor cortex of participants during the observation of abstract artworks from Lucio Fontana (Umilta' et al., 2012) and from Franz Kline (Sbriscia-Fioretti et al., 2013). These experiments demonstrated that the intrinsic dynamism of the observed abstract artworks activates the motor simulation of the artist's gesture in the brain of beholders. In addition, a very recent transcranial magnetic stimulation (TMS) study using a set of pictures of Umberto Boccioni's sculpture “Unique Forms of Continuity in Space” (1913) revealed that beholding of these very dynamic static images increased the excitability of beholders' cortical motor system (Concerto et al., 2015).

Given the results of our behavioral experiment, the next step will be to replicate it while recording EEG activity with the hypothesis that titles manipulation should modulate not only the explicit evaluation of the perceived dynamism, but also the excitability of the observers' sensorimotor cortex. We expect an impact of the title upon the motor simulation of the intrinsic dynamism characteristic of Futurists', leading to increase the activation of beholders sensorimotor cortex.

## Author contributions

Both authors designed the study. Testing and data collection were performed by SM. MU performed the data analysis. Both authors interpreted the results. SM drafted the manuscript. Both authors wrote and approved the final version of the manuscript.

## Funding

The research was funded by the Department of Education, University of Roma Tre.

### Conflict of interest statement

The authors declare that the research was conducted in the absence of any commercial or financial relationships that could be construed as a potential conflict of interest.

## Appendix

Title of the artworks and their manipulations

1. Giacomo Balla, *Girl running on a Balcony*, 1912, Museo del Novecento, Milano (original title, *La bambina che corre sul balcone*).
       Neutral Title: *Girl on a Balcony*
       Increased Title: *Girl running fast on a Balcony*
2. Giacomo Balla, *Dynamism of a Dog on a Leash*, 1912, Albright-Knox Art Gallery, Buffalo, NY, USA (original title, *Dinamismo di un cane al guinzaglio*).
       Neutral Title: *Dog on a Leash*
       Increased Title: *Fast Dynamism of a Dog on a Leash*
3. Giacomo Balla, *Flight of swallow*, 1913, Private collection (original title, *Volo di rondini*).
       Neutral Title: *Swallow*
       Increased Title: *Fast flight of swallow*
4. Giacomo Balla: *Expansion x velocity* 1913. Milano, Civica Galleria d'Arte Moderna, Raccolta Grassi (original title, *Espansione x velocità*).
       Neutral Title: *Automobile*
       Increased Title: *Expansion x high velocity*
5. Giacomo Balla, 1913, *Automobile* + *velocity* + *light*, Collezione Jucker (original title, *Automobile* + *velocità* + *luce*).
       Neutral Title: *Automobile*
       Increased Title: *Automobile* + *high velocity* + *light*
6. Umberto Boccioni, 1911, *Running train*, Private collection (original title, *Treno in corsa*).
       Neutral Title: *Train*
       Increased Title: *Fast Train running*
7. Benedetta Cappa Marinetti, *Velocity of a boat*, 1919, Roma, Galleria Comunale di arte moderna e contemporanea (original title, *Velocità di motoscafo*).
       Neutral Title: *Boat*
       Increased Title: *High Velocity of a boat*
8. Carlo Carrà, 1910, *Dynamism of swimmers*, Carnegie Institute, Pittsburg, USA (original title, *Dinamismo di nuotatrici*).
       Neutral Title: *The swimmers*
       Increased Title: *Fast dynamism of swimmers*
9. Luigi Russolo, *Dynamism of an automobile*, 1912-1913. Parigi, Musèe National d'Art Moderne, Centre Georges Pompidou (original title, *Automobile in corsa*).
       Neutral Title: *Automobile*
       Increased Title: *Fast dynamism of an automobile*
10. Gino Severini, 1912, *Dynamism of a dancer*. Pinacoteca di Brera, Milano (original title, *Dinamismo di una danzatrice*).
       Neutral Title: *Dancer*
       Increased Title: *Fast dynamism of a dancer*
